# Assessment of the Left Ventricular Dysfunction in Patients With Acromegaly Using Global Longitudinal Strain by Two-Dimensional Speckle Tracking Echocardiography and Tissue Doppler Imaging

**DOI:** 10.7759/cureus.78936

**Published:** 2025-02-13

**Authors:** Dhanachand S Nameirakpam, Anupama Hegde, Himamshu Acharya, Pramila Kalra, Arun S Moirangthem

**Affiliations:** 1 Cardiology, Regional Institute of Medical Sciences, Imphal, IND; 2 Cardiology, M S Ramaiah Medical College, Bengaluru, IND; 3 Endocrinology and Metabolism, A.J. Institute of Medical Sciences, Mangaluru, IND; 4 Endocrinology and Metabolism, M S Ramaiah Medical College, Bengaluru, IND; 5 Biostatistics, Regional Institute of Medical Sciences, Imphal, IND

**Keywords:** 2-dimensional echocardiography, acromegaly complications, global longitudinal strain, growth hormone excess, left atrial volume index, left ventricular mass index, left ventricular systolic dysfunction, speckle tracking echocardiography

## Abstract

Introduction

Acromegaly is a rare disease resulting from excess growth hormone (GH) and insulin-like growth factor-1 (IGF1), with cardiovascular complications being frequently encountered, leading to increased morbidity and mortality. The aim of the study was to determine the frequency of left ventricular systolic dysfunction in patients with acromegaly using global longitudinal strain (GLS) by 2D speckle tracking echocardiography and also the frequency of left ventricular diastolic dysfunction by tissue Doppler imaging (TDI).

Materials and method

A cross-sectional study involving 20 acromegaly patients with normal left ventricular (LV) systolic function as measured by ejection fraction and 20 controls with age, sex, and comorbidities matched were included in the study from 2021 to 2023. All these patients underwent 2D speckle tracking echocardiography to assess GLS and TDI with conventional 2D Echocardiography to assess diastolic function.

Results

GLS was significantly lower in the acromegaly group, which was -15.79±2.54 (mean±SD), than in the control group, which was -17.47±0.98 (mean±SD) with p < 0.05, indicating significant LV systolic dysfunction in the acromegaly group. The majority of the acromegaly group had abnormal GLS (n=11; 55%). The majority of the acromegaly patients with increased left atrial volume index (LAVi) had abnormal GLS (n=8/11; 72.7%). Also, the majority of the acromegaly patients with increased LVMi had abnormal GLS (n=8/12; 66.66%). TDI study for diastolic dysfunction showed no significant difference between the acromegaly group and the control group (p > 0.05). LAVi in the acromegaly group was 31.35±6.22 (mean±SD), and in the control group was 27.00±4.81(mean±SD) with p < 0.05, which was statistically significant. LAVi was more in the active acromegaly group (4 males and 2 females) than inactive acromegaly group (3 males and 2 females). LVMi in the acromegaly group was 100.32±24.335 (mean±SD), and in the control group, it was 85.85±19.63 (mean±SD) with p < 0.05, indicating more LV hypertrophy. LVMi was more in the active (5 females and 4 males) than the inactive acromegaly group (2 females and 1 male). Statistical significance was observed in LVID between the acromegaly group, which was 4.75±0.52 (mean±SD), and the control group, which was 4.41±0.49 (mean±SD) with a p < 0.05. Septal medial early diastolic velocity (e` med) in the acromegaly group was 0.08±0.03 (mean±SD), and in the control group was 0.10±0.02 (mean±SD) with p < 0.05, which was statistically significant. The multiple linear regression analysis revealed that acromegaly, hypertension, and higher body surface were the most important predictors of abnormal GLS.

Conclusions

Abnormal GLS indicating subclinical LV systolic dysfunction in patients with acromegaly can be evaluated by 2D speckle tracking echocardiography. Active acromegaly patients had more abnormal GLS, increased LAVi, and increased LVMi than inactive acromegaly patients. LV diastolic dysfunction was not remarkable when the acromegaly group and control group were assessed as the comorbidities were matched. The presence of acromegaly, hypertension, and higher body surface area had a significant negative effect on GLS.

## Introduction

Acromegaly is a rare condition resulting from growth hormone (GH) and insulin-like growth factor-1 (IGF-1) excess. The altered GH/IGF-1 axis in acromegaly results in various cardiovascular, respiratory, and metabolic complications. In the majority of the cases with acromegaly, GH is secreted by a pituitary adenoma. GH/IGF1 axis regulates myocyte growth and structure, cardiac contractility, and vascular function. The action of GH/IGF-1 excess results in characteristic biventricular hypertrophy. GH has both direct and indirect action through IGF-1 on the cardiovascular system. GH directly acts on the myocardium by increasing myocardial sensitivity to calcium. GH also increases cardiac collagen deposition. IGF-1 promotes collagen synthesis by fibroblasts. Of the cardiovascular consequences of acromegaly, hypertension and left ventricular (LV) hypertrophy are the most common, but serious valvular regurgitation and heart failure can also develop [1].

LV strain is a marker of early and subclinical ventricular dysfunction. Strain describes the deformation of the myocardium that occurs during the cardiac cycle in the longitudinal, circumferential, and radial planes. It offers a more sensitive study of myocardial contractility [2]. Speckle tracking echocardiography (STE) can be used to evaluate the global longitudinal strain (GLS), which reflects LV function and may help to identify subclinical LV systolic dysfunction. STE has an advantage over conventional 2D echocardiography in the measurement of myocardial deformation or strain. Speckle tracking involves analysis of the motion of speckles in the 2D ultrasonic image. Reductions in GLS portend worse cardiovascular outcomes [3].

Tissue Doppler imaging (TDI) assists in the judgment of diastolic function by quantifying myocardial velocities in multiple segments from different echocardiographic acoustic windows. TDI can detect diastolic dysfunction (DD) in patients with active acromegaly, even when LV muscle mass is normal or slightly elevated [4]. In our study, the conventional 2D echocardiographic method was also used to assess LAVi and LVMi.

Aim

The aim of this study was to assess LV dysfunction by GLS using 2D STE and TDI with conventional 2D Echocardiography in acromegaly patients with normal ejection fraction (EF) in comparison with the control group. Our study involved a comparison of subjects with acromegaly and the control group without this rare disease, which was matched in terms of age, sex, and comorbidities. This study will be beneficial in assessing the role of STE in identifying the individual at risk at an early stage, especially when the standard echo parameters such as EF are normal.

## Materials and methods

Study population

This was a cross-sectional study. Twenty patients (10 males and 10 females; mean age 44.5 ± 7.48) with acromegaly (Active disease - 13 and Inactive disease - 7 ) were included in this study after applying the inclusion and exclusion criteria. As age, gender, diabetes, and hypertension can affect echocardiographic parameters, these were matched when recruiting controls. The data from all 20 acromegaly patients were matched to those of the control population in terms of age, sex, and comorbidities (diabetes, hypertension, or both). The control group consisted of 20 subjects (10 males and 10 females; mean age 44.5 ± 7.48). Five patients (4 males and 1 female) were excluded from the study due to LV systolic dysfunction from the acromegaly group. Three patients (2 males and 1 female) were also excluded due to poor 2D echocardiographic image quality, which will lead to discrepancies in measuring the strain value. Patients aged from 18 years to 60 years with both active and inactive acromegaly were enrolled in the study. Patients with a history of coronary artery disease, systolic heart failure, stroke/transient ischemic attack, chronic kidney disease, and pregnancy were excluded from both groups. The study was conducted at Ramaiah Medical College Hospital and Ramaiah Memorial Hospital, tertiary care hospitals in Bengaluru, Karnataka, a southern Indian state.

The diagnosis of acromegaly was based on current clinical standards - typical clinical features, unsuppressed GH level (GH ≥ 1 ng/mL) 2 hours post 75 g oral glucose tolerance test (OGTT) or elevated IGF-1 levels for age and positive pituitary MRI findings [5]. The acromegaly group was further divided based on the serum level of GH and/or IGF-1 concentration. For treated cases, if the level of IGF-1 was above the age and sex-specific threshold or if the GH level was ≥ 1 ng/mL post-OGTT, the disease was considered to be active [5,6].

Systolic and diastolic blood pressure was measured for all subjects and they were categorized as hypertensive if blood pressure (BP) was above 140/90 mmHg [7].

Diagnosis of diabetes mellitus was based on the American Diabetes Association (ADA) criteria, diagnosed when the fasting plasma glucose level is ≥126 mg/dL in two consecutive measurements when the glucose level is ≥200 mg/dL two hours after an OGTT, or random blood sugar > 200mg/dl with symptoms, or HbA1c ≥ 6.5% [8].

When a subject is receiving treatment for previously diagnosed diabetes mellitus, plasma glucose, and HbA1c were measured.

Echocardiography

2D-Echocardiography

All the study subjects underwent two-dimensional echocardiography using a high-quality echocardiograph (Vivid E9, GE, USA). A sector transducer with a frequency of 1.7 to 3.3 MHz was used. All the study subjects underwent 2D echocardiography evaluation with all the standard views according to American Society of Echocardiography (ASEC) Chamber Quantification guidelines.

Analysis by Standard 2D Echocardiography

LV diastolic function was assessed using pulse wave Doppler, tissue Doppler index, LV mass index ((LVMi), and LV volume index.

Using pulse wave Doppler, LV diastolic function was measured using the formula E/A, where E is the mitral E-wave velocity and A is the mitral A-wave velocity. The E-wave refers to early LV diastolic filling velocity, and the A-wave refers to late LV diastolic filling velocity [9]. Tissue Doppler indices E' med and E/E' were also used to assess DD. E' med refers to the septal mitral annulus early diastolic velocity and is measured in centimeters per second (cm/s). E/E' is the ratio of mitral E-wave velocity to mitral annular velocity [9].

LVMi was calculated using M-mode. The following parameters were measured in the parasternal long axis view (PLAX view): - LV internal dimension in end-diastole (cm, LVIDd); interventricular septal thickness in end-diastole (cm, IVSTd), and posterior wall thickness in end-diastole (cm, PWTd). LV mass (LVM) was determined using the formula of the ASEC modified by Devereux [10]. LVMi was then calculated by indexing the LVM to the body surface area (BSA). LV hypertrophy was defined as an LVMi of >115 g/m² in men and >95 g/m² in women [9].

LAVi was calculated using the biplane area-length method. The volume was determined by tracing the inner border of the left atrium (LA), excluding the area under the mitral valve (MV) annulus, pulmonary veins, and LA appendage, from the apical 4-chamber and 2-chamber views. Using the area-length technique, the length (L) was measured from the shorter of the two long axes in the 4C and 2C views. The left atrial volume was then calculated using the formula.

LA Volume = (8/3)π × (A₁ × A₂) / L = 0.85 × (A₁ × A₂) / L

A1 - Maximum planimetered LA area in apical 4-chamber (A4C) view

A2 - Maximum planimetered LA area in apical 2-chamber (A2C) view

L - Length measured from back wall to line across mitral valve hinge points (cm)

Then, the LAV was indexed to BSA for the LAVi [9].

Ejection fraction for left ventricular systolic function

The calculation was done using the Simpson method [9]. Visual estimation was used when imaging was difficult by two highly qualified specialists in echocardiography [11].

Two-dimensional speckle tracking echocardiography

All the study subjects underwent speckle tracking techniques using Vivid E9 BT 13 version with GE EchoPAC software. Left ventricular GLS was measured by 2D STE to measure LV systolic function. GLS is defined as the change in the length of an object within a certain direction relative to its baseline length. It is a measurement of deformation that is used to assess LV systolic function. Strain (%) = (Lt - Lo) / Lo (Lt is the length at time t, Lo is the length at time 0). GLS describes the relative length change of the LV myocardium between end-diastole and end-systole: GLS (%) = (MLs - MLd)/MLd, where ML is the myocardial length at end-systole (MLs) and end-diastole (MLd). Because MLs are smaller than MLd, peak GLS is a negative number [9]. Because of the short acquisition time, feasibility, accuracy, and repeatability, automated function imaging (AFI) was used to assess LV function [12]. Images from the apical four-chamber, two-chamber, and three-chamber views were acquired in succession. During imaging, ECG gating was done to acquire a high-quality ECG signal which made proper gating of the images. During measurement, it was made sure that the heart rate variation was less than 5 bpm [13]. A high temporal resolution of 50-60 frames per second was acquired to ensure acoustic marker tracking. For the timing of aortic valve opening and closure, a continuous wave (CW) of the aortic valve or pulse wave (PW) of the left ventricular outflow tract (LVOT) was obtained. By tracking the displacement of the speckles during the cardiac cycle, strain and the strain rate were measured offline; after adequate image acquisition. During the analysis, only the images with appropriate tracking in all the myocardial segments were used. GLS value was the average of the value that was obtained for three apical views. The reference range for LV-GLS was -24% to -16% [14]. So, lesser GLS values below 16 were considered abnormal.

Statistical analysis

The statistical analysis was carried out with SPSS 20.0 software (IBM, USA). Normal distribution was confirmed with the Shapiro-Wilk and Kolmogorov Smirnov test before data were analyzed. Appropriate tests were used for further analysis depending on the distribution of variables. Student’s t-test was used to compare the average values of two variables with a normal distribution (for paired data or depending on the situation). Multiple linear regression analysis was performed to determine which variables were predictors of GLS. Statistical significance was established for p-values less than 0.05.

## Results

Demographic and clinical characteristics of the study group

There was no significant difference observed in BMI and BSA in the acromegaly group and control group. Age, sex, and comorbidities were matched and, hence, were similar in both disease and control groups, as shown in Table 1.

**Table 1:** Demographic and clinical characteristics of patients with acromegaly and of controls. BMI: Body mass index; BSA: Body surface area

	Acromegaly Group (n =20)	Control Group (n=20)	P-Value
Age (Years)	44.5 ± 7.48	44.5 ± 7.48	1.000
Sex (Female/Male)	10/10	10/10	1.000
BMI (Kg/m^2^)	26.12 ± 3.16	25.59 ± 2.98	0.581
BSA (m^2^)	1.80 ± 0.19	1.79 ± 0.15	0.857
Hypertension (Yes/No)	10/10	10/10	1.000
Diabetes (Yes/No)	8/12	8/12	1.000
Active acromegaly	13/20	Not applicable	

Left ventricular global longitudinal strain by 2D speckle tracking echocardiography

There was a statistically significant difference in GLS between the acromegaly group, which was -15.79±2.54 (mean±SD), and the control group, which was -17.47±0.98 (mean±SD) with p-value < 0.05 as shown in Table 2 which signifies LV systolic dysfunction in acromegaly group as compared to controls despite having normal EF.

Abnormal global longitudinal strain (GLS)

Out of 20 patients in the acromegaly group, 9 (45%) had normal GLS, while the remaining 11 (55%) exhibited abnormal GLS (Table 2). The acromegaly group had significantly increased LAVi, among which the majority had abnormal GLS (n=8/11; 72.7%). The acromegaly group also had significantly increased LVMi, among which the majority had abnormal GLS (n=8/12; 66.66%).

**Table 2:** 2D Echocardiography parameters in patients with acromegaly versus the control group IVSTs: Interventricular septal thickness in systole; IVSTd: Interventricular septal thickness in diastole; PWTs: Posterior wall thickness in systole; PWTd: Posterior wall thickness in diastole; LVMi: LV mass index; LAVi: Left atrial volume index; E/A (cm/s): Early (E) to Late (A) diastolic mitral inflow velocity ratio; e` med: Medial early diastolic velocity; E/e`: Ratio of early diastolic mitral inflow velocity to early diastolic mitral annular velocity; EF: Ejection fraction; GLS: Global longitudinal strain

	Patients with Acromegaly (n=20)	Control Group (n=20)	P-Value
Cardiac Chamber Size			
LVIDs (cm)	3.09±0.35	2.98±0.44	0.375
LVIDd (cm)	4.75±0.52	4.41±0.49	0.041
LV Mass			
IVSTs (cm)	1.26±0.19	1.28±0.13	0.595
IVSTd (cm)	1.05±0.18	1.02±0.13	0.537
PWTs (cm)	1.29±0.20	1.34±0.18	0.491
PWTd (cm)	1.05±0.21	1.03±0.14	0.725
LVMi (g/m^2^)	100.32±24.335	85.85±19.63	0.045
LAVi (mL/m^2^)	31.35±6.22	27.00±4.81	0.018
Diastolic Function			
E/A	0.96±0.42	1.00±0.32	0.735
e` med (cm/s)	8±3	10±2	0.024
E/e`	9.46±3.56	7.99±2.35	0.132
Systolic Function			
EF (%)	58.7±2.81	60.0±3.60	0.211
GLS ((%)	-15.79±2.54	-17.47±0.98	0.009

Comparison of abnormal GLS between acromegaly and control groups

There was a significant difference in the proportion of normal/abnormal GLS in the acromegalic and control groups, as shown in Table 3.

**Table 3:** Comparison between the acromegaly and control groups GLS: Global longitudinal strain; LVMi: LV mass index; LAVi: Left atrial volume index

	Patients with Acromegaly (n=20)	Control	P-Value
GLS		
Normal	9 (45.00%)	20 (100.00%)	
Abnormal	11 (55.00%)	-	
Diastolic function			>0.05
Normal	6 (30.00%)	11 (55.00%)	
Abnormal	14 (70.00%)	9 (45.00%)	
LAVi			<0.001
Normal	7 (35.00%)	18 (90.00%)	
Abnormal	13 (65.0%)0	2 (10.00%)	
LVMi			<0.001
Normal	8 (40.00%)	16 (80.00%)	
Abnormal	12 (60.00%)	4 (20.00%)	

Comparison of GLS of active and inactive acromegaly patients with controls

In comparison with the active GLS and control group, the active GLS group had significantly decreased GLS (15.12 ± 2.47 vs. 17.47 ± 0.98; p < 0.001), highlighting remarkable LV systolic dysfunction in this group (Table 4). However, no significant difference was observed in the GLS between the inactive and the control group (17.04 ± 2.33 vs. 17.47 ± 0.98; p = 0.498).

**Table 4:** GLS comparison in active and inactive acromegalic patients and controls GLS: Global longitudinal strain (expressed in absolute values)

GLS (%)	N	Mean ± SD	P-Value
Active GLS vs Control			<0.001
Active GLS	13	15.12 ± 2.47	
Control	20	17.47 ± 0.98	
Inactive GLS vs Control			0.498
Inactive GLS	7	17.04 ± 2.33	
Control	20	17.47 ± 0.98	

Two-dimensional echocardiographic evaluation of the study groups

On echocardiographic evaluation (Table 2), there was a statistical significance observed in LVIDd between the acromegaly group, which was 4.75±0.52 (mean±SD), and the control group, which was 4.41±0.49 (mean±SD) with a p < 0.05. LAVi in the acromegaly group was 31.35±6.22 (mean±SD), and in the control group was 27.00±4.81(mean±SD) with p < 0.05, which was statistically significant. LAVi was higher in males (7) than females (4) in the acromegaly group. LAVi was more in the active (4 males and 2 females) than the inactive acromegaly group (3 males and 2 females). LVMi in the acromegaly group was 100.32±24.335 (mean±SD), and in the control group, it was 85.85±19.63 (mean±SD) with p < 0.05 which was statistically significant, indicating more LV hypertrophy. LVMi was more in females (7) than males (5). LVMi was more in the active (5 females and 4 males) than the inactive acromegaly group (2 females and 1 male). Septal medial early diastolic velocity (e` med) in the acromegaly group was 0.08±0.03 (mean±SD), and in the control group was 0.10±0.02 (mean±SD) with p < 0.05, which was statistically significant. The rest of the parameters showed no significant difference. In the TDI study for DD, no statistical significance was observed between the acromegaly groups and control groups with p > 0.5, as shown in Table 3. Comparison of LAVi showed statistical significance with p < 0.001, which highlights a significant difference in the proportion of normal/abnormal LAVi in acromegalic and control groups as shown in Table 3. Comparison of LVMi showed statistical significance with p< 0.001, highlighting the significant difference in the proportion of normal/abnormal LVMi in acromegalic and control groups as shown in Table 3.

Multiple linear regression analysis

The regression analysis revealed that patients having acromegaly, hypertension, and higher BSA had a significant negative effect on GLS with R2 value =0.337 as shown in Table 5 (F-value = 6.102, p-value = 0.002).

**Table 5:** Multiple linear regression (MLR) model summaries for prediction of GLS as dependent variable GLS: Global longitudinal strain

Predictor Variables	Coefficient	Std. Error	95% CI		t-value	P-value
			Lower Limit	Upper Limit		
(Constant)	24.263	3.009	18.162	30.365	8.065	0.000
Acromegaly	-1.65	0.558	-2.782	-0.518	-2.955	0.005
Hypertension	-1.406	0.563	-2.549	-0.263	-2.496	0.017
Body surface area	-3.391	1.632	-6.701	-0.082	-2.078	0.045

Model Fit Statistics

R² = 0.337, F-value = 6.102, overall p-value = 0.002

## Discussion

The presence of any cardiovascular disease at the time of diagnosis of acromegaly indicates a significant increase in the risk of death, and the mortality rate may even reach 100% within 15 years [15]. The common cardiovascular complications in patients with acromegaly mainly include hypertension, acromegalic cardiomyopathy, valvular disease, and arrhythmia [15,16]. The main features of acromegalic cardiomyopathy are ventricular concentric hypertrophy and impaired diastolic function. In the terminal stages, both systolic and diastolic function may decrease due to extracellular collagen deposition [17].

In this study, we observed the clinical utility of GLS measurement by STE in detecting subclinical LV systolic dysfunction in acromegaly patients with normal LVEF. To date, this is the first such report/study with age, sex, and comorbidities matched in patients with acromegaly (both active & inactive) and controls. A study done by Popielarz-Grygalewicz et al. comparing the LV function of 26 subjects with acromegaly with 52 controls revealed a significantly lower GLS in acromegaly (-16.6% vs. -20.7%; p < 0.01) [18]. In our study, we found that abnormal GLS were more common in the acromegaly group compared to the control group, showing LV systolic dysfunction despite having a normal EF. The mean GLS in the acromegaly group was -15.79±2.54 (mean±SD), and the control group which was -17.47±0.98 (mean±SD) with p = 0.009, which was similar to the previous study done by Popielarz-Grygalewicz et al. There was a significant difference in the proportion of normal/abnormal GLS in acromegaly and control group. We also found that the acromegaly group had significantly increased LAVi, of which the majority had abnormal GLS (n=8/11; 72.7%). The acromegaly group also had significantly increased LVMi, among which the majority had abnormal GLS (n=8/12; 66.66%). Among acromegaly patients, 13 (65%) had active disease and 7 (35%) had inactive disease. We also observed that GLS in active acromegaly patients was significantly lower (abnormal) compared to controls (-15.12% vs. -17.47%, P < 0.001). Despite statistical significance, it should be interpreted with caution as our active acromegaly group was heterogeneous, including treatment-naive cases, postoperative cases, and individuals on somatostatin receptor ligand (SRL) therapy. Another study by Popielarz-Grygalewicz et al. observed that the median GLS showed a significant improvement after three months of SRL treatment compared to baseline values, with GLS changing from -20.0% to -20.4% (p=0.045) [19]. This finding underscores the potential utility of STE in scenarios where traditional echocardiographic parameters lack the sensitivity to detect differences.

LVDD develops in the early stages of acromegaly. Guo et al. observed that DD was common (50.5%) in acromegaly patients (OR = 15.62, 95% CI = 1.98-123.34) [20]. In this study, we found that, in the TDI study for DD, no statistical significance was observed between the acromegaly and control groups. This lack of statistical significance is potentially due to the matching of comorbidities in our study. In a previous study, Sicolo et al. observed that although acromegalic cardiomyopathy leads to abnormal ventricular relaxation causing DD, contractility and EF tend to remain unchanged [21]. In our study, although we could find significant differences in GLS, we could not find significant differences in DD parameters such as E/A and E/e'. Hypertension and diabetes are independently associated with impaired LV diastolic function, regardless of the effect of overweight/obesity and other covariates. Their coexistence results in a negative synergistic effect on LV diastolic mechanics and is associated with higher LV filling pressures than either condition alone [22]. These findings contribute to understanding the lack of statistical significance in our study of LVDD.

LAVi significantly predicts mortality risk, independent of LV geometry, and adds to the overall mortality prediction in a large cohort of patients with preserved systolic function, as revealed by Patel et al. [23]. Acromegaly patients were at higher risk for atrial fibrillation, congestive heart failure, and all-cause death [24]. Increased LAVI is associated with cardioembolic stroke as well as AF detection in patients with embolic stroke of undetermined source [25]. According to these findings assessment of LAVi is necessary in patients with acromegaly with conventional 2D echocardiography. Popielarz-Grygalewicz et al. indicated that nearly 80% of acromegaly patients exhibit increased LAVi. In contrast, LVH is observed in less than 50% of patients, suggesting that adaptive LA remodeling takes precedence over LV remodeling in the cardiac progression of acromegaly patients [18]. In this study, we also found that 65% of the acromegaly patients had increased (abnormal) LAVi. LAVi in the acromegaly group was 31.35±6.22 (mean±SD), and in the control group was 27.00±4.81(mean±SD) with p < 0.001, which was statistically significant. LAVi was more prevalent among males (7) than females (4) in the acromegaly group. LAVi was more in the active (4 males and 2 females) than in the inactive acromegaly group (3 males and 2 females). Also, comparing LAVi, there was a significant difference in the proportion of normal/abnormal LAVi in the acromegaly and control groups. This study also found that most acromegaly patients with increased LAVi had abnormal GLS (n=8/11; 72.7%).

A study done by Uziȩbło-Życzkowska et al. revealed that when compared to the control group, the acromegaly group had higher LVM (LVMi: 132 vs. 108 g/m2, p < 0.001), more frequent LV hypertrophy (80.0 vs. 53.3%; p = 0.028) [26]. Here in this study, we also observed that LVMi in the acromegaly group was 100.32±24.335 (mean±SD) and the control group, which was 85.85±19.63 (mean±SD) with p < 0.05, indicating more LV hypertrophy. LVMi was increased (abnormal) in 60% of patients with acromegaly. LVMi was more in females (7) than males (5). LVMi was more in active (5 females and 4 males) than the inactive acromegaly group (2 females and 1 male). When comparing LVMi, there was a significant difference in the proportion of normal/abnormal LVMi in the acromegaly and control group with p < 0.001, as shown in Table 3. Increased LVMI and IGF-1 were strongly associated with decreased LV-GLS in acromegaly patients [27]. Koca et al. observed that every 1 ng/dL increase in IGF-1 level increased the probability of decreased LV-GLS by 5.6% [28]. Similarly, in this study, most acromegaly patients with increased LVMi had abnormal GLS (n=8/12; 66.66%). Therefore, intervention in hypertension, diabetes, and LVMI as early as possible may be the most logical way to delay future systolic dysfunction in patients with acromegaly.

In this study, we also observed that on echocardiographic evaluation (Table 2), LVIDd between the acromegaly group, which was 4.75±0.52 (mean±SD), and the control group, which was 4.41±0.49 (mean±SD) with a p < 0.05 was significant. Septal e` med in the acromegaly group was 0.08±0.03 (mean±SD), and in the control group was 0.10±0.02 (mean±SD) with p < 0.05, which was significant. The rest of the parameters showed no significant difference, which again may be attributed to our study as comorbidities were matched.

In this study, multiple linear regression (MLR) analysis was also performed, which revealed that patients with acromegaly, hypertension, and an increase in BSA had a significant negative effect on GLS. Adequate treatment of hypertension, disease control by surgery or SRL therapy might have a beneficial effect on GLS in patients with acromegaly.

Clinical implications

GLS by 2-dimensional STE analysis allows assessment of impaired subclinical LV systolic dysfunction in patients with acromegaly, both active and inactive despite having normal LVEF. Active acromegaly patients had more abnormal GLS and increased LAVi and LVMi compared to inactive acromegaly. Patients with acromegaly and hypertension had a significant negative effect on GLS. This novel method will be helpful in the early identification of individuals with acromegaly at risk, which will serve as a key to implementing early treatment to prevent and reduce the cardiovascular morbidity and mortality associated with acromegaly.

Limitations

The limitation of this study is its small sample size. Another limitation of this study was the presence of both clinically active and inactive disease groups among subjects with acromegaly. Because of the rarity of the disease, we could not avoid choosing both active and inactive diseases in our study. We could not include IGF1 or GH levels in the linear regression model, especially for the active acromegaly subjects, as levels at the time of echocardiography were not available for all the patients, and categorization of clinical activity was based on the last available GH/IGF1 levels.

## Conclusions

Abnormal GLS indicating subclinical LV systolic dysfunction in patients with acromegaly can be evaluated using 2D STE. Active acromegaly patients exhibited more abnormal GLS, increased LAVi, and increased LVMi compared to the control group. LVDD was not notable when comparing the acromegaly group to the control group, as comorbidities were matched. Patients with acromegaly and hypertension showed a significant negative impact on GLS. This study underscores the importance of STE in identifying subclinical LV dysfunction in individuals with acromegaly.
